# Sudden loss of visual acuity following intra-articular steroid injection in to the knee joint: a case report

**DOI:** 10.1186/1757-1626-1-428

**Published:** 2008-12-30

**Authors:** Saikrishna Balakrishnan, Sunil Apsingi, Sanjiv B Manjure

**Affiliations:** 1 Department of Orthopaedics & Trauma, Level 4, Centre Block, Colney Lane, Norfolk & Norwich University Hospital NHS Trust, Norwich, NR4 7UU, UK; 2 Trauma & Orthopaedics, Luton & Dunstable NHS Trust, Lewesey Road, Luton, LU4 0DZ, UK

## Abstract

**Background:**

Exogenous and endogenous steroids have been implicated in the pathogenesis of central serous choroioretinopathy in the literature. Central serous choroioretinopathy presents as sudden reduction in visual acuity.

**Case presentation:**

Despite the fact that steroid injections are so commonly used in the Orthopaedics there is no information regarding central serous chorioretinopathy in Orthopaedic literature. We report a case of acute onset central serous chorioretinopathy following an intra-articular injection.

**Conclusion:**

Orthopaedic surgeons and doctors dealing with Musculoskeletal problems need to be aware of this unusual complication of systemic steroid therapy and refrain from administering further steroids to patients with history of blurring of vision or sudden loss of visual acuity following previous steroid injections.

## Case presentation

A 59-year-old female patient American Society of Anesthesiologist grade 1(ASA 1) underwent an arthroscopy of right knee for a suspected meniscal tear. Degenerative tears of the medial meniscus and grade III osteoarthritic changes in the medial compartment of the knee were found. Partial medial menisectomy was performed and, 80 mg of methylprednisolone acetate (Depo-Medrone^® ^Pharmacia Ltd, UK) and 10 ml of 0.5% bupivacaine were injected into the joint at the end of the procedure through the arthroscope under direct visualization. Thirty six hours after the surgery our patient developed sudden blurring of vision in her both eyes. She was referred urgently to the acute eye clinic, where she was found to have decreased visual acuity of 6/60 bilaterally. Fundus examination showed subretinal precipitate with central serous choroidal retinopathy. She was advised to avoid any type of steroids and was followed up in the outpatient Ophthalmology department. At the last Ophthalmology appointment three months after the incident she had improved symptomatically, visual acuity was bilaterally 6/9. Fundus examination revealed resolving central serous choroidal retinopathy. She was discharged from the eye clinic.

## Discussion

Central serous chorioretinopathy is characterized accumulation of subretinal fluid at the posterior pole of the fundus, creating a circumscribed area of serous retinal detachment [1,2,4,5]. There is an increased incidence of central serous chorioretinopathy in conditions associated with high endogenous [2,6] or exogenous steroid levels. Exogenous corticosteroids administered by various routes such as oral, inhalation, epidural & intra-articular [3,5,7] have been shown to be associated with central serous chorioretinopathy. The pathogenesis of central serous chorioretinopathy remains incompletely understood. It is Garg et. Al [2] reported to be a multi-factorial condition with the steroids playing a pivotal role in pathogenesis. Some of the various postulated mechanisms include inhibition of collagen synthesis, increasing choriocapillaries permeability and ion pumping dysfunction with a reversal of ionic current direction [4,7,8]. Central serous chorioretinopathy usually resolves spontaneously in majority of the patients [7,8]. Though good central visual acuity is restored in most of the patients they may still notice loss of color vision, loss of contrast sensitivity, image distortion or nyctalopia [7]. They have a risk of recurrence, resulting in progressive retinal atrophy and permanent visual loss. Our patient presented with visual symptoms thirty six hours after the methylprednisolone acetate injection into knee joint. Bovine and equine intra-articular methylprednisolone acetate injections have resulted in a pharmacologically significant raise in concentration of methylprednisolone causing such complication. We were alerted to this by the Ophthalmologist and were advised not administer any more steroids. Our patient subsequently recovered her visual acuity. Mondal et al [7] have described the only other case of bilateral central serous choroidal retinopathy following intra-articular steroid injection. Their patient received intra-articular triamcinolone acetonide and presented with bilateral impairment of vision on the next day. Our patient presented thirty six hours after the injection. Orthopaedic surgeons and doctors dealing with Musculoskeletal problems need to be aware of this unusual complication of systemic steroid therapy and refrain from administering further steroids to patients with history of blurring of vision or sudden loss of visual acuity following previous steroid injections.

## Consent

Written informed consent was obtained from the patient for publication of this case report and accompanying images. A copy of the written consent is available for review by the Editor-in-Chief of this journal.

## Competing interests

The authors declare that they have no competing interests.

## Authors' contributions

SB was involved in writing the paper with data collection and literature search

SA, Registrar was involved in treating the patient and data collection

SM, Consultant was involved in treating the patient
